# Bilateral Facial Nerve Palsy: A Diagnostic Dilemma

**DOI:** 10.1155/2012/458371

**Published:** 2012-01-23

**Authors:** Sohil Pothiawala, Fatimah Lateef

**Affiliations:** Department of Emergency Medicine, Singapore General Hospital, Outram Road, Singapore 169608

## Abstract

*Introduction*. Bilateral facial nerve palsy (FNP) is a rare condition, representing less than 2% of all cases of FNP. Majority of these patients have underlying medical conditions, ranging from neurologic, infectious, neoplastic, traumatic, or metabolic disorders. *Objective*. The differential diagnosis of its causes is extensive and hence can present as a diagnostic challenge. Emergency physicians should be aware of these various diagnostic possibilities, some of which are potentially fatal. *Case Report*. We report a case of a 43-year-old female who presented to the emergency department with sequential bilateral facial nerve paralysis which could not be attributed to any particular etiology and, hence, presented a diagnostic dilemma. *Conclusion*. We reinforce the importance of considering the range of differential diagnosis in all cases presenting with bilateral FNP. These patients warrant admission and prompt laboratory and radiological investigation for evaluation of the underlying cause and specific further management as relevant.

## 1. Introduction

Unilateral facial nerve palsy (FNP), with an incidence of around 25 per 100,000 population, is a common neurologic disorder mimicking a stroke. It often leads to emergency department visits. Bell's palsy, also known as idiopathic facial paralysis, is the most common cause of unilateral facial paralysis, accounting for approximately 70% of these cases [1].

Bilateral FNP is exceedingly rare, representing less than 2% of all the facial palsy cases, and has an incidence of 1 per 5,000,000 population [2, 3]. Majority of these patients have serious underlying medical conditions and need to be admitted for evaluation of the underlying cause and further management. Bell's palsy accounts for only 23% of bilateral facial paralysis [1].

We report a case of a 43-year-old female who presented to our department with sequential bilateral facial paralysis, in which unilateral FNP was followed by contralateral FNP in the next 2 days before complete resolution of symptoms on the side affected first. We also discuss her evaluation and the possible differential diagnoses. As few cases have been reported, the literature review serves as an important point of discussion for bilateral facial palsy.

## 2. Case Report

A 43-year-old lady with no previous medical illness presented to the emergency department with complaints of perioral numbness, altered tongue sensation, speech difficulty, and facial droop as noted by her husband since that morning. She was able to mobilize well with no peripheral limb weakness. She had been complaining of upper back pain for the previous 3 days and was seen by a general practitioner and given symptomatic medications with not much relief. She had mild headache and vomiting on the day before her presentation. She denied any fever, headache, neck ache, visual disturbances, giddiness, or tinnitus. There was no history of trauma, travel in the last few years, or joining any adventure expeditions. She denied any history of smoking, alcohol intake, blood transfusion, or sexual promiscuity.

Examination revealed right facial nerve (lower motor neuron) palsy with mild slurring of speech. Examination of the other cranial nerves was normal. Power in all limbs was normal, deep tendon reflexes were present, and her sensory examination was unremarkable. There were no cerebellar signs.

Computed tomography of the brain was done which did not reveal any obvious abnormality. She was referred to the ophthalmology department and no exposure keratopathy was identified. The patient was presumptively diagnosed to have right Bell's palsy and discharged with acyclovir, prednisone, analgesia, eye patch, and moisturizing eye drops. She was given an outpatient otolaryngology followup.

She represented to the emergency department after 3 days with progressive bilateral weakness of the face and drooling. She also had some blurred vision then but denied any weakness of limbs. Examination revealed bilateral lower motor neuron facial nerve palsy. No other neurological sign was elicited during this presentation. Her hematological and biochemical blood tests, including liver function tests and thyroid levels, were normal.

As bilateral facial palsy is suggestive of a possible underlying etiology, the patient was admitted under the care of neurology department. Chest X-ray and spinal X-rays were reported to be normal. Patient declined lumbar puncture and CSF examination. Serological tests for various agents, including thyroid peroxidase antibodies, antinuclear antibody, anti neutrophil cytoplasmic antibody, syphillis antibody, lyme (Borrelia) IgM, and Epstein-Barr virus capsid Ag IgM antibody tests, were all negative. Lyme (Borrelia) IgG and Epstein-Barr virus capsid Ag IgG antibody tests were positive. Magnetic resonance imaging (MRI) of the brain was reported to have bilateral asymmetrical enhancement of the facial nerves, right more than left, at the apex of their intracanalicular portion, as well as their labyrinthine, tympanic, and mastoid segments, which is may be due to atypical Bell's Palsy.

There was no progression of her symptoms during her admission. She was discharged in a stable condition after a 3-day stay in the hospital and she completed the course of acyclovir and prednisone. The patient has been asymptomatic since then and no recurrence has been noted during her followup visits at the National Neurology Institute.

## 3. Discussion

Bilateral facial nerve palsy is a rare condition and hence presents a diagnostic challenge. Unlike the unilateral presentation, it is seldom secondary to Bell's palsy. The majority of patients with bilateral facial palsy have Guillain-Barre Syndrome (GBS), multiple idiopathic cranial neuropathies, Lyme disease, sarcoidosis, meningitis (neoplastic or infectious), brain stem encephalitis, benign intracranial hypertension, leukemia, Melkersson-Rosenthal syndrome (a rare neurological disorder characterized by facial palsy, granulomatous cheilitis, and fissured tongue), diabetes mellitus, human immunodeficiency virus (HIV) infection, syphilis, infectious mononucleosis, malformations as Mobius Syndrome, vasculitis, or bilateral neurofibromas. The possibility of intrapontine and prepontine tumor should also be considered [2, 4, 5]. Thus, it should be carefully investigated before establishing the diagnosis of Bell idiopathic palsy [6].

The most common infectious cause of bilateral FNP is Lyme disease, caused by spirochete *Borrelia burgdorferi*, whose carrier is a common tick [7]. Bilateral FNP can be seen in about 30–35% of patients with Lyme disease. Diagnosis is serologic, and IgM antibodies increase in the second week and tend to decrease with treatment, while IgG antibodies appear late with reaching its peak in the second or third month, and it can indefinitely remain positive [8, 9]. Hence, they cannot be used to distinguish active from inactive disease. As in our case, the IgM antibody against this organism in serum was negative, but IgG antibody was positive. This would suggest that the patient might have had an infection at some time in the past and hence it could not be related as a cause of the clinical symptoms. Lyme disease is not prevalent in Singapore [10]. Moreover, as the patient did not present with any recent history of contact with ticks or travel to endemic areas, the diagnosis was less likely.

Guillain-Barre Syndrome (GBS) is an inflammatory postinfectious polyradiculoneuritis of uncertain etiology. Bilateral FNP can occur in up to 50% of the fatal cases. The diagnosis is made on clinical findings of peripheral areflexia, and lumbar puncture shows liquoric dissociation: elevated protein in the absence of raised cell number [2, 4, 8]. Our patient refused to have a lumbar puncture but did not have any peripheral weakness or areflexia. Hence, GBS was excluded as an underlying etiology.

Traumatic skull fractures and cerebello-pontine angle tumors were excluded by CT and MRI of the brain. Sarcoidosis was excluded as there was no hilar adenopathy reported on chest X-ray. Diabetes has been noted to be present in 28.4% of patients with bilateral FNP and can be explained by the fact that diabetic patients are more prone to nerve degeneration [11, 12]. Normal blood sugar levels excluded diabetes as a cause of bilateral FNP in our patient.

Approximately 40% of the Epstein-Barr virus- (EBV- ) infection-associated facial nerve palsy cases are bilateral [13]. It has been well documented that acute EBV infections in the pediatric age group cause FNP, but few cases have been reported in the adult population [14]. Positive EBV serological tests have been reported in patients with Bell's palsy. Most cases of EBV infectious mononucleosis are subclinical, and the only manifestation of EBV infection is a serological response to EBV surface proteins discovered with EBV serological tests. Our patient had a negative Epstein-Barr virus capsid antigen (VCA) IgM antibody test, but VCA IgG antibody test was positive. She had no clinical manifestations of EBV infection. EBV IgM VCA titers decrease in most patients after 3–6 months but may persist in low titer for up to 1 year. EBV IgG VCA antibodies rise later than the IgM VCA antibodies but remain elevated with variable titers for life. Increased IgG VCA indicates past exposure to EBV, which may have been subclinical or clinical. Also, increased IgG VCA titers are not synonymous with chronic infectious mononucleosis [15]. Moreover, FNP may be an initial manifestation of Non-Hodgkin's lymphoma (NHL), which would result if EBV-induced B-lymphocyte proliferation is uncontrolled. Thus, excluding an acute EBV infection should be considered in patients presenting with bilateral FNP, although it is rare.

There has been a report of brain MRI finding in bilateral Bell's palsy, which showed abnormal bilateral enhancement of the proximal intracanalicular segments but no facial nerve swelling [16]. Enhancement of the facial nerve is nonspecific and may relate to either hypervascularity of the perineural structures of the nerve or actual disruption of the blood-nerve barrier. Enhancement of the intracanalicular portion of the facial nerve should be considered abnormal [17]. MRI is superior to CT Brain in evaluating each segment of the facial nerve at various levels. MRI would also be useful to detect neoplasms compressing the seventh cranial nerve or cerebello-pontine angle tumors. Contrast-enhanced MRI scan if done in the appropriate clinical setting may detect a positive radiographic diagnosis of Bell's palsy which had been previously considered a diagnosis of exclusion [18]. Hence, an MRI scan of the brain should be considered as the modality of choice for radiological evaluation of FNP.

Differential diagnosis mandating further investigation is very important because the treatment and prognosis depends on the cause. Although bilateral FNP may show more severe paralysis, the overall prognosis in most cases is as good as that in unilateral FNP, excluding life-threatening or traumatic cases [19]. Recovery is similar to that in unilateral palsy, although one side of the face may recover earlier than the other [20].

Thus, our case of bilateral FNP presented a diagnostic dilemma and the definite etiology could not be ascertained.

## 4. Conclusion

Unilateral facial palsy is usually idiopathic or related to viral illness. On the other hand, bilateral facial nerve palsy is a rare, diagnostically challenging presentation. Emergency physicians should be aware of the various diagnostic possibilities, some of which are life-threatening and potentially fatal. We reinforce the importance of considering the range of differential diagnosis in all cases presenting with bilateral facial nerve palsy. These patients need thorough assessment and warrant admission and prompt laboratory and radiological investigation for evaluation of the underlying cause and specific further management as relevant.
